# Effects of Fluoride and Calcium Phosphate Materials on Remineralization of Mild and Severe White Spot Lesions

**DOI:** 10.1155/2019/1271523

**Published:** 2019-06-16

**Authors:** Zixiang Dai, Min Liu, Yansong Ma, Li Cao, Hockin H. K. Xu, Ke Zhang, Yuxing Bai

**Affiliations:** ^1^Department of Orthodontics, School of Stomatology, Capital Medical University, Beijing, China; ^2^Department of Preventive Dentistry, School of Stomatology, Capital Medical University, Beijing, China; ^3^Department of Endodontics, Periodontics and Prosthodontics, University of Maryland Dental School, Baltimore, MD 21201, USA

## Abstract

Fixed orthodontic treatments often lead to enamel demineralization and cause white spot lesions (WSLs). The aim of this study was to evaluate the mineralization degree of 2 types of WSLs based on ICDAS index and compare the remineralizing efficacy of 3 oral hygiene practices after 1 month and 3 months. 80 mild demineralized and 80 severe demineralized enamel specimens were randomized into three treatments: fluoride toothpaste (FT), fluoride varnish plus fluoride toothpaste (FV+FT), and CPP-ACP plus fluoride toothpaste (CPP-ACP+FT). Microhardness tester, DIAGNODent Pen 2190, and scanning electron microscope were used to evaluate the changes of mineralization degree. Both qualitative and quantitative indicators suggested that the mild and severe white spot lesions were different in the degree of mineralization. Severe WSLs demineralized much more seriously than mild lesions even after 3 months of treatment. Despite the variation in severity, both lesions had the same variation trend after each measure was applied: FT had weak therapeutic effect, FV + FT and CPP-ACP + FT were effective for remineralization. Their remineralizing efficacy was similar after 1 month, and combined use of CPP-ACP plus F toothpaste was more effective after 3 months. In order to fight WSLs, early diagnosis was of great importance, and examination of the tooth surface after air-dry for 5 seconds was recommended. Also, when WSLs were found, added remineralizing treatments were required.

## 1. Introduction

Enamel demineralization is one of the most undesired side effects of fixed orthodontic treatment [1, 2]. The brackets, bands, and wires and other attachments interfere in effective oral hygiene procedure, causing plaque gathering, which exacerbates the risk of demineralization [3]. Studies have shown that white spot lesions (WSLs) can develop within 1 month after bonding [4]. The prevalence of WSLs after orthodontic treatment varies from 2% to 96% [2, 5]. Enamel demineralization not only violates the aesthetic principle of orthodontic treatment but also damages tooth health. Besides all the active preventative methods, early and accurate detection of WSLs and effective remineralizing treatments are also crucial.

International Caries Detection and Assessment System (ICDAS) index is a visual detection system based on evidence-based medicine and has been acknowledged internationally; it enables detailed detection and assessment of dental caries with different degrees of the developing progress [6]. From the index, white spot lesions could be classified as score 1 and score 2. Score 1 means an early visual change in enamel. When seen wet, there is no evidence of any change in color that can be attributed to carious activity, but after prolonged air-drying for 5 seconds, a carious opacity is visible. Score 2 means a distinct visual change in enamel when viewed wet [7]. Studies have shown that the ICDAS index has great consistency, sensitivity, repeatability, and great correlation with histology examination [8–10]. It was reported that the ICDAS index was the best way to detect changes in enamel, comparing with bitewing radiographs, laser fluorescence devices, and fluorescence camera devices [10].

There are many kinds of materials for enamel remineralization at present, which can be categorized into 3 groups: (1) Professionally applied agent, like fluoride varnish and fluoride foam. These agents have the advantage of being independent of patient compliance and are quick and simple for dentists to apply. Duraphat (Colgate, CA, USA) is one of the most commonly used fluoride varnishes, which contains 5% sodium fluoride. (2) Self-applied agents under the guidance of dentists: casein phosphopeptide-amorphous calcium phosphate (CPP-ACP) agent, for instance. These materials require close cooperation from the patients. MI Paste plus (GC, Tokyo, Japan) contains 10% CPP-ACP and 900 ppm fluoride. (3) Daily oral hygiene practice agent, like fluoride toothpaste, normally contains 0.00%-0.14% fluoride. Hence, routine oral hygiene practice, routine oral hygiene practice plus professionally applied agent, and routine oral hygiene practice plus self-applied agent under the guidance of dentists are 3 common ways for fighting WSLs.

Topical use of fluoride has become a widely recognized method for enamel remineralization since Schmidt applied fluoride varnish to orthodontic patients firstly in 1964 [11]. The formation of insoluble fluorapatite and calcium fluoride increases the enamel resistance for demineralization. When saliva pH decreases, bonded calcium ion could release, promoting remineralization [12, 13].

CPP-ACP is a bioactive agent derived from milk products, which can stabilize supersaturated calcium ion and phosphorus ion in the oral environment. When pH decreases, stabilized ions would release; phosphorus ion buffers the pH condition, and calcium ion promotes remineralization [14]. The remineralizing ability has been proved by both in vitro and in vivo studies [15–19]. In addition to the remineralizing capacity itself, study showed that, despite stabilizing and transforming calcium and phosphate ion, CPP is also a good carrier of fluoride ion, which could transform fluoride ion into deeper lesion area, enhancing its permeability. As a result, the increasing fluoride ion concentration generates more fluorapatite and hydroxyapatite, promoting more remineralizing effects [20].

The remineralization effects of both fluoride varnish and CPP-ACP agent on WSLs have been proved previously. Some studies demonstrate that CPP-ACP is superior to fluoride varnish for remineralization [16, 17], while other studies indicate that fluoride varnish is more effective [14, 21, 22]. The discrepancy in the literature could be possibly attributed to the variations in study design, treatment duration, differences agents, and the including criteria of lesions.

However, when WSLs are found in clinic practice, the lesions have already reached score 2, at least. At present, few studies have focused on the degree of mineralization of mild and severe WSLs and the prognosis of remineralization therapy for different measures. Since score 1 means an earlier stage of WSL, would the early visual diagnosis be helpful for treatment? Is the prognosis of mild lesions better than that of severe ones? Would the remineralizing efficacy differ when the lesion's severity varied?

Therefore, the aim of the study was to (1) establish and evaluate the mineralization degree of 2 types of WSLs models based on ICDAS in vitro and (2) compare the remineralizing efficacy of fluoride toothpaste; fluoride varnish +F toothpaste; CPP-ACP + F toothpaste on mild and severe WSLs.

## 2. Materials and Methods

### 2.1. Specimen Preparation

Premolars extracted for orthodontic purpose were selected, and the selection criteria included intact buccal and lingual enamel surfaces without WSLs, without visible cracks or enamel irregularities. The use of extracted human teeth has been approved by the Ethical Committee of Beijing Stemmatological Hospital. All the teeth were cleaned and stored in 0.1% thymol solution at 4°C before use within 1 month.

For each sample, the root was cut off and the crown was buccolingually sectioned into two parts using a water-cooled diamond bur; then crown segments were embedded in a self-curing acrylic resin (New Century Dental Materials, Shanghai, China) exposing the most prominent part of enamel. Each sample was polished with 600 to 2000 grit abrasive papers under flooding water. To define the experimental area, the central area was painted with acid-resistant nail varnish, leaving a 3 × 3 mm^2^ space of exposed enamel [23].

### 2.2. Testing Procedure

#### 2.2.1. Hardness Measurement

Vickers hardness (HV) was determined by using digital hardness tester (FM-700, Future Tech, Tokyo, Japan). Five indentations were conducted in different regions of each specimen randomly using a square based diamond pyramid Vickers's indenter under a load of 50 g for 15s [24]. Then the average value was calculated.

#### 2.2.2. DIAGNOdent Pen Reading

DIAGNOdent Pen 2190 (Kavo, Biberach, Germany) was used to assess the surface change presented on the experimental window. As recommended by the manufacturer, the device was calibrated using the standard ceramic supplied by the manufacturer before every measurement. Type B probe, which was recommended for smooth surface detection, was used. The device was held perpendicular to the test site and rotated along the lesion to collect the fluorescence from all directions. Each sample was tested 3 times; then the average value was calculated and recorded [25].

#### 2.2.3. Scanning Electron Microscope

The specimens were cleaned with an ultrasonic cleaner and examined via a scanning electron microscope (Phenom-world Co., LTD., Netherlands, SEM). Surface topography of the specimens was obtained.

### 2.3. Lesion Creation (Demineralization)

**Figure 1:**
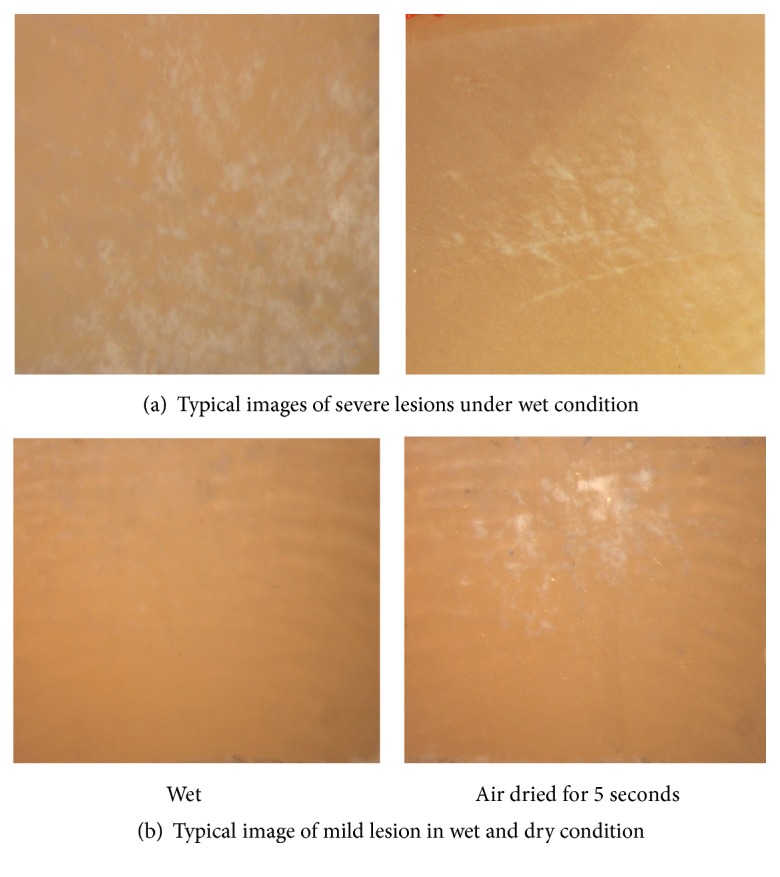


The demineralization solution consisted of 2.2 mmol/L CaCl_2_, 2.2 mmol/L Na_2_HPO_4_, and 50 mmol/L HAC, and the pH was adjusted to 4.4 by 1 mmol/L NaOH [26]. The specimens were immersed in the solution at 37°C. Every day, visual detection was conducted by 2 clinicians from orthodontic department and preventative dentistry department under the clinic light. The examiners had received training from the online learning program released by ICDAS organization on its website (https://www.iccms-web.com/content/iccms-usage/clinical-practice), which included a theoretical part and evaluation of projected images of carious teeth; the Kappa value of consistency test for two inspectors after practice was 0.7. During the detection, if WSLs could be observed under wet condition, which was in accordance with ICDAS score 2, the sample would be sorted into “severe lesions” group and transferred in distill water. If the window was intact, the sample would be dried with compressed air for 5 seconds and re-evaluated under dry condition. If WSL could be observed then, which was in accordance with ICDAS score 1, the sample would be sorted into “mild lesions” group and then transferred in distill water. Generally, mild lesions could be observed in 2-4 days; severe lesions took about 6 days to be detected. If no WSLs were found, the sample will be immersed back to the demineralization solution. Typical images of lesions were taken with 10X magnified visual field of stereomicroscope (Olympus Corporation, Tokyo, Japan) (Figure 1). The detection was implemented separately. If the two clinicians had the same score, then it was valid. If scores were inconsistent with each other, the specimen would be immersed back into the demineralization solution and re-evaluated on the next day.

The procedure was repeated until 80 mild demineralized and 80 severe demineralized enamel specimens were collected. Hardness, DIAGNOdent Pen readings, and SEM images of the 2 groups were detected.

### 2.4. Remineralizing

Artificial saliva solution was prepared by dissolving, in distilled water (0.4 g/L NaCl, 0.4 g/L KCl, 0.795 g/L CaCl_2_·H_2_O, 1 g/L Urea, and 0.005 g/L Na_2_S·2H_2_O, 0.78 g/L) NaH_2_PO_4_·H_2_O [27]. Under the same severity, four groups were established based on the remineralizing method:

(1) Artificial saliva (referred to as Control);

(2) Fluoride toothpaste (Colgate, CA, USA) group (referred to as FT);

(3) Fluoride varnish (Duraphat, Colgate, CA, USA) plus F toothpaste (referred to as FV + FT);

(4) CPP-ACP (MI Paste plus, GC, Tokyo, Japan) plus F toothpaste (referred to as CPP-ACP + FT).

Each group contained 10 specimens. The treatment duration was 1 month and 3 months.

For Control group, all the specimens were followed by the pH cycling model as described later.

For FT group, specimens were brushed with a cotton swab dipped with pea-size fluoride toothpaste for 5 seconds, imitating the Bass Method, then rinsed with distill water for 5 seconds, and immersed back into the artificial saliva. The procedures were conducted twice a day, once in the morning at 7:30 and once in the evening at 21:00.

For FV + FT group, on the first day of 1-month-treatment group, on the first day and in the beginning of the sixth week of the 3-month-treatment group [28, 29], each specimen was brushed with a thin layer of Duraphat and then dried for 4 hours and immersed in the artificial saliva. From the second day, each specimen was brushed with fluoride toothpaste as group 2 described.

For CPP-ACP + FT group, after toothbrushing procedure as group 2 described, a thin layer of MI Paste plus was applied and left undisturbed for 30 mins on tooth surfaces and then the specimens are rinsed with distill water for 5 seconds and immersed back into the artificial saliva. This procedure was applied according to the manufacturer's instruction. MI Paste plus was implemented 2 times a day, immediately after tooth brushing.

### 2.5. pH Cycling Model

After food taken, oral bacteria decomposed sugar and produce acid, causing pH decline. In order to imitate the oral environment in vitro, besides the remineralizing process, all the specimens were immersed in the demineralization solution for 3 hours and in artificial saliva for the rest 21 hours every day. The 3-hour-demineralization time consists with the total pH decrease time after 3 meals. This procedure is called “pH cycling” [30, 31].

**Figure 2:**
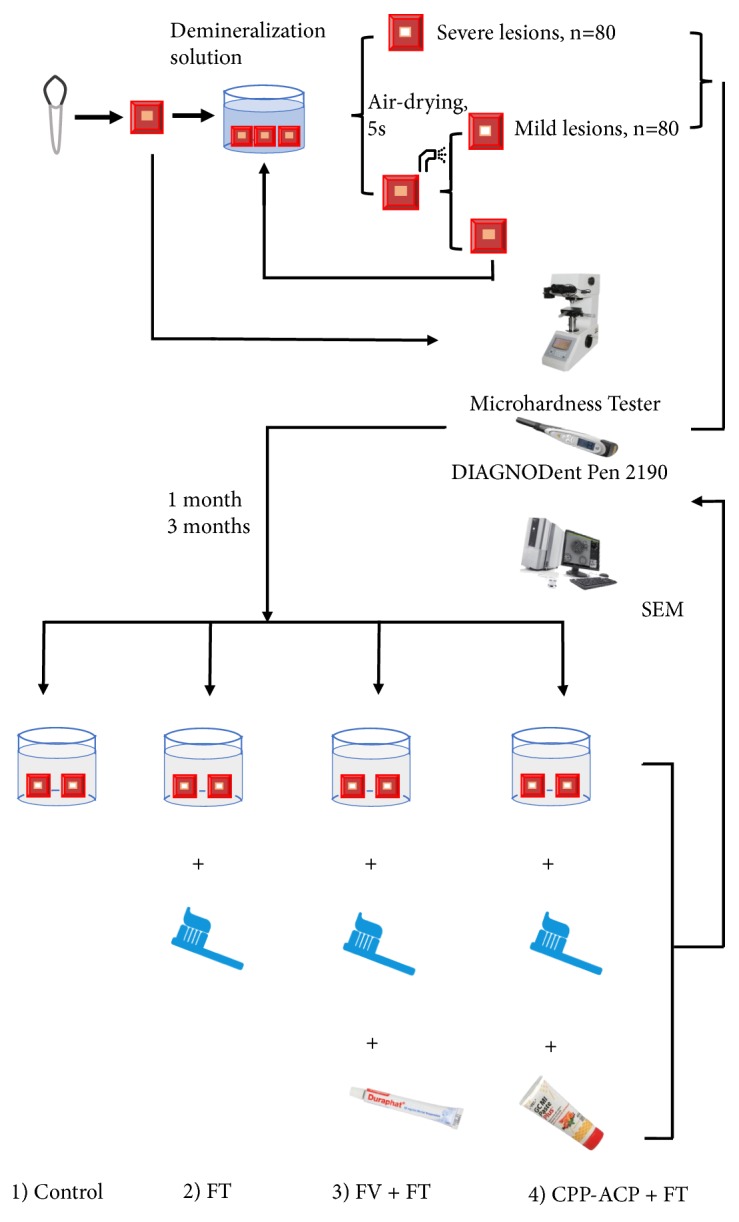
The experimental procedure of this study.

After the completion of each treatment duration, specimens were detected by microhardness tester, DiagnoDent Pen 2190 and SEM, and then stored in distill water at 4°C. The experimental procedures were shown in Figure 2.

### 2.6. Statistical Analysis

Enamel hardness and DIAGNODent Pen reading results were statistically examined with SPSS 17.0 for Windows (SPSS Inc. Chicago, IL, USA). The normal distribution assumption and Levene's homogeneity test were confirmed for the dependent variables. Independent t-test and ANOVA with Tukey post hoc tests were used. The significance level of p was set at 0.05.

## 3. Results

**Figure 3:**
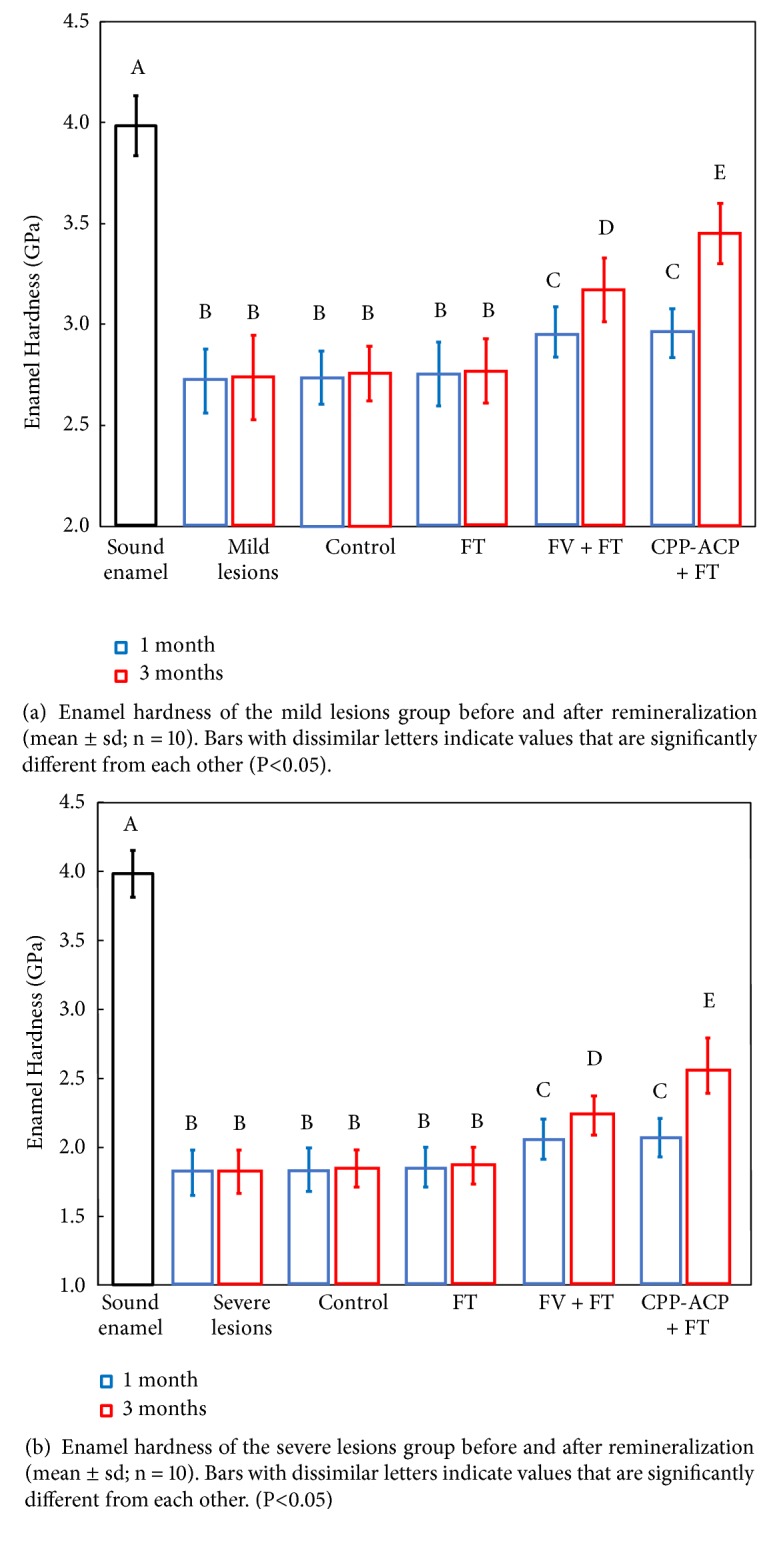


The results of enamel hardness were shown in Figure 3. For mild demineralization groups (Figure 3(a)), the value of “Control, 1m” group, “Control, 3m” group, “FT, 1 m” group, and “FT, 3 m” group increased, but there was no significant difference between them and with that after demineralization (P > 0.05). Enamel hardness of “FV + FT, 1 m” group and “CPP-ACP + FT, 1 m” group increased significantly comparing with the 3 groups above (P<0.05), and there was no statistic difference between those two groups (P > 0.05). The value of “CPP-ACP + FT, 3 m” group was the highest, which was statistically different from the other groups (P<0.05). For severe demineralization groups (Figure 3(b)), the variation trend of enamel hardness was consistent with that of mild demineralization groups.

**Figure 4:**
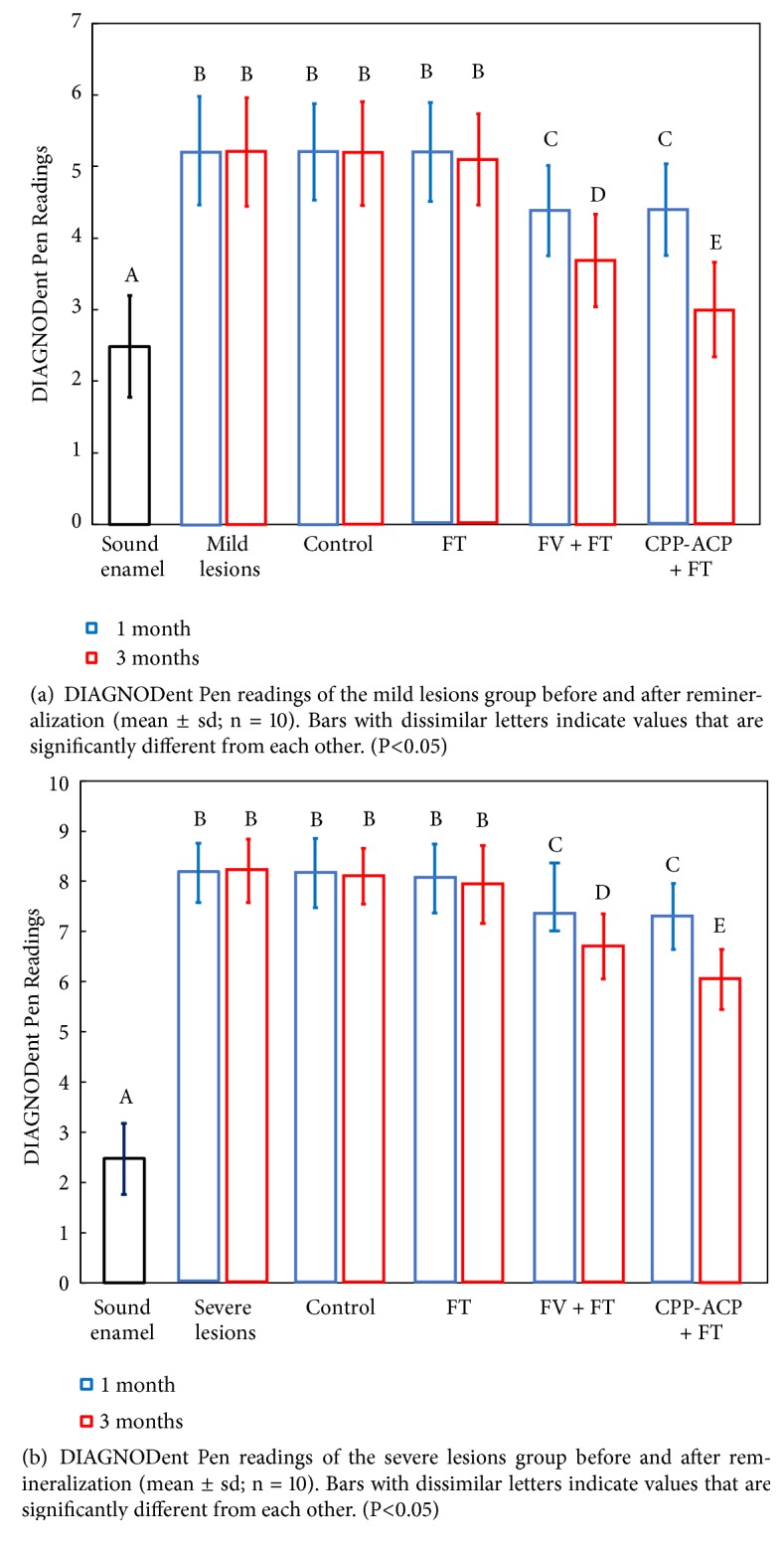


DIAGNODent Pen readings of different stages of enamel specimens were shown in Figure 4. For mild demineralization groups (Figure 4(a)), no statistical difference was found among the readings of “Control, 1m” group, “Control, 3m” group, “FT, 1 m” group, and “FT, 3 m” group and with that after demineralization (P > 0.05). Readings of “FV + FT, 1 m” group and “CPP-ACP + FT, 1 m” group were at the same level (P > 0.05), which were significantly decreased comparing with the 3 groups above (P<0.05). The reading of “CPP-ACP + FT, 3 m” group was the lowest, which was statistically different from the other groups (P<0.05). For severe demineralization groups (Figure 4(b)), the variation trend of DIAGNODent Pen readings was in accordance with that of mild demineralization groups.

**Figure 5:**
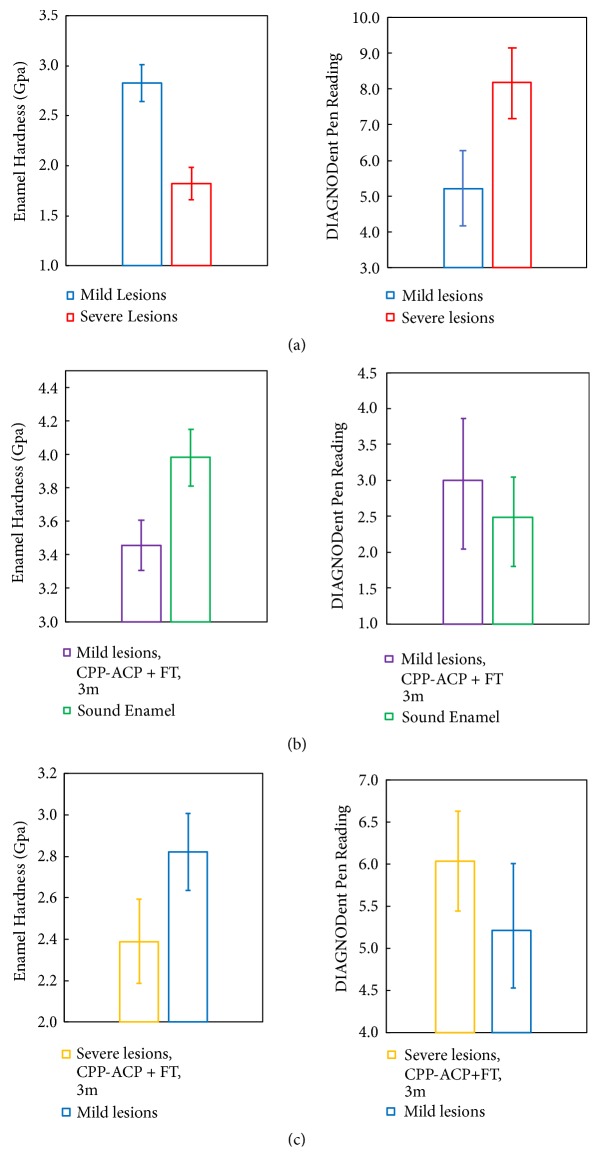
Typical intergroup comparison of enamel hardness and DIAGNODent Pen 2190 readings. Statistic difference could be found in every graph. (P<0.05).

Figure 5 showed the typical intergroup comparison.  Figure 5(a) was the comparison between mild lesions and severe lesions, and both enamel hardness and DIAGNODent Pen readings suggested that severe lesions were much more seriously demineralized (P<0.05). Statistically differences (P<0.05) could be found in the comparison between sound enamel and the “CPP-ACP + FT, 3 m” group (which represented the strongest therapeutic effect in this study) of mild lesions, as Figure 5(b) showed. In Figure 5(c), mild demineralized specimens and the “CPP-ACP + FV, 3 m” group of severe lesions were compared. Results suggested that their degree of mineralization was statistically different from each other (P<0.05).

**Figure 6:**
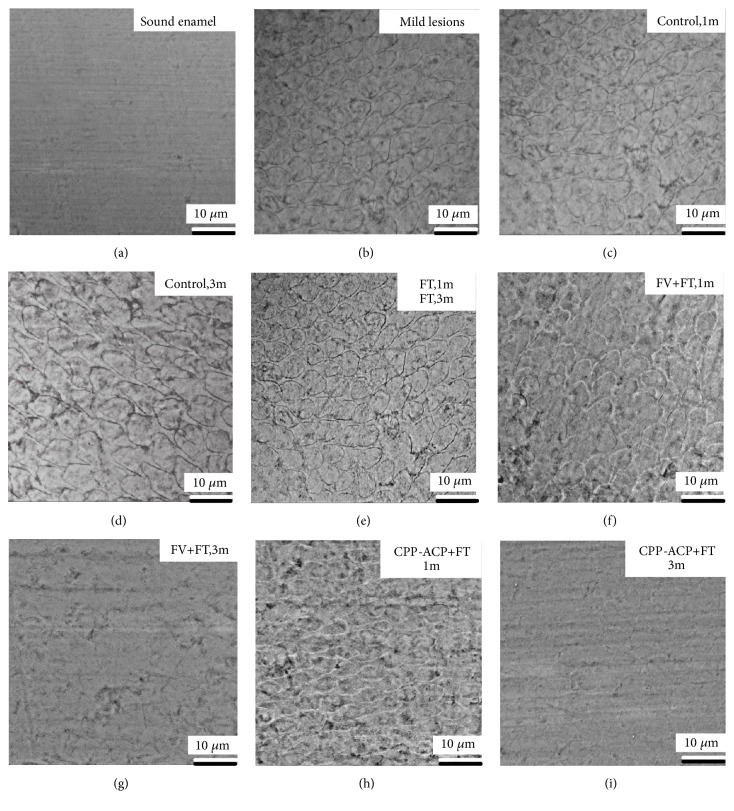
The typical SEM image of mild lesions of each group. (a) Sound enamel (before demineralization); (b) mild lesions (after demineralization); (c) artificial saliva immersion for 1 month (Control, 1 month); (d) artificial saliva immersion for 3 months (Control, 3 months); (e) fluoride toothpaste brushing for 1 month and 3 months (FT, 1 m; FT, 3 m); (f) combined use of fluoride varnish plus fluoride toothpaste for 1 month (FV + FT, 1 month); (g) combined use of fluoride varnish plus fluoride toothpaste for 3 months (FV + FT, 3 months); (h) Combined use of CPP-ACP plus fluoride toothpaste for 1 month (CPP-ACP + FT, 1 month); (i) combined use of CPP-ACP plus fluoride toothpaste for 3 months (CPP-ACP + FT, 3 months).

**Figure 7:**
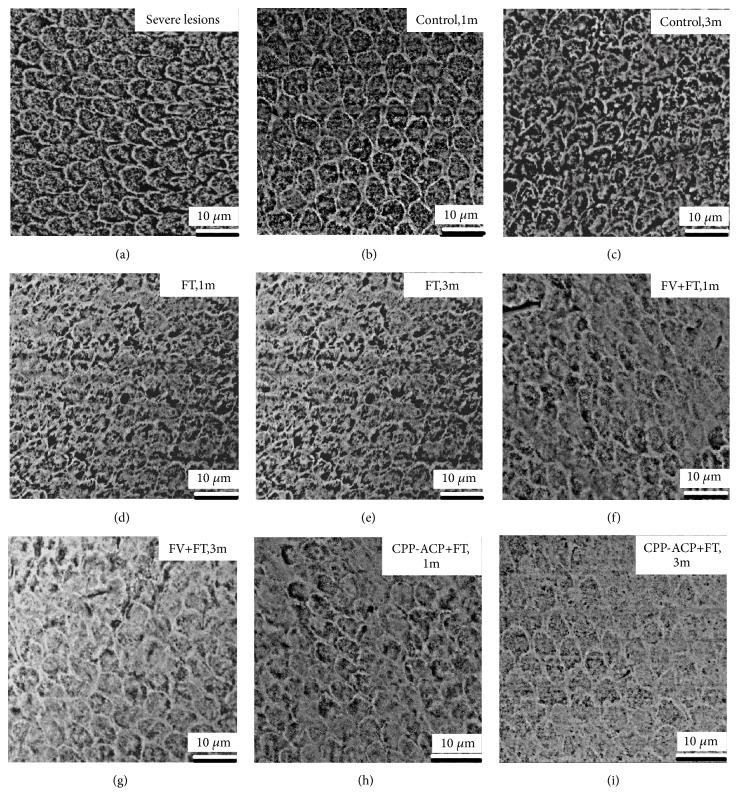
The typical SEM image of severe lesions of each group. (a) Severe lesions (after demineralization); (b) artificial saliva immersion for 1 month (Control, 1 month); (c) Artificial saliva immersion for 3 months (Control, 3 months); (d) fluoride toothpaste brushing for 1 month (FT, 1 month); (e) fluoride toothpaste brushing for 3 month (FT, 3 months); (f) combined use of fluoride varnish plus fluoride toothpaste for 1 month (FV + FT, 1 month); (g) combined use of fluoride varnish plus fluoride toothpaste for 3 months (FV + FT, 3 months); (h) combined use of CPP-ACP plus fluoride toothpaste for 1 month (CPP-ACP + FT, 1 month); (i) combined use of CPP-ACP plus fluoride toothpaste for 3 months (CPP-ACP + FT, 3 months).

Typical SEM images of the enamel surface topography for each group were presented in Figures 6 and 7. As Figure 6(a) depicted, before demineralization, most of the enamel surface was smooth and flat, and small cracks, clefts, pores, and transverse lines can be seen in some places. For mild lesions group, the SEM image of mild lesions, “Control, 1 m” group, “Control, 3 m” group, “FT, 1 m group”, and “FT, 3 m group (Figures 6(b), 6(c), 6(d), and 6(e)) looked similar: the surface of enamel was rough, severe demineralization took place around the enamel sheath, and shallow elliptical clefts could be observed. The prism demineralized partly, and the pit-like pores were visible. The image of FT group at 1 month was similar to that of the FT group at 3 months and hence was not included to avoid redundancy. The SEM image of “FV + FT, 1 m” group and “CPP-ACP + FT, 1 m” group (Figures 6(f) and 6(h)) looked similar: minerals deposited on the enamel surface, forming a partially flat surface. But prism boundaries and localized depressions in the prism were still obvious. From the image of “FV + FT, 3 m” group (Figure 6(g)), mineral deposit increased significantly. The restored enamel surface became smoother. However, there were still a few images of enamel sheath and pores left. For “CPP-ACP + FT, 3 m” group, enamel surface looked smooth and flat. The edge of the prism was dimly visible, leaving only a few enamel sheath and pores, as shown in Figure 6(i). For severe lesions groups, the SEM image of severe lesions, “Control, 1 m” group, “Control, 3 m” group, “FT, 1 m group”, and “FT, 3 m group looked similar (Figures 7(a), 7(b), 7(c), 7(d), and 7(e)): the enamel surface looked rough, and dissolution of both prism and interprismatic substance were severe. Deep elliptical clefts and large holes could be observed. The SEM image of “FV + FT,1m” group (Figure 7(f)) and “CPP-ACP + FT, 1 m” group (Figure 7(h)) looked similar: minerals deposit formed small area of flat surface, and holes in the prisms reduced. A large number of wide clefts and deep pores were still invisible. After 3-month FV + FT treatment, mineral deposit increased. More areas of smooth enamel surface could be seen. Deep clefts and large holes became smaller pores, as Figure 7(g) showed. After 3-month CPP-ACP + FV treatment, mineral deposits were significant. Enamel surface was further leveled. Large pores in the prism disappeared mostly. Pores with smaller diameters were still common, as showed in Figure 7(i).

## 4. Discussion

This study established two types of white spot lesions model in vivo according to ICDAS index and evaluated the mineralization degree of them. The results of both quantitative and qualitative measurements after demineralization were in accordance with the visual feature. This was the same as Ekstrand's finding [32], who correlated the severity of carious lesions to their histological depth: white spot lesions which require air-drying were most likely to be limited to the outer half of the enamel, while the depth of white spot lesions which was obvious without air-drying was located in the inner half.

Previously, the assessment of remineralization efficacy did not take the impact of the lesion condition into account. According to the result, the severity of mild and severe lesions was statistically different. The variation trend of remineralization was similar and the prognosis after remineralizing treatment varied.

After remineralization, although test indicators rose, statistic results suggested that artificial saliva and fluoride toothpaste were inefficient for treating white spot lesions. Some clinical research found that, after debonding, only by using fluoride toothpaste could the mineral condition be improved and the area of WSLs be decreased [25]. However, long-term follow-up showed that although the area decreased, it did not disappear completely but stabilized in a certain area [33]. This phenomenon is mainly due to the decrease of enamel hardness after demineralization. Brushing and masticatory movement caused abrasion of the tooth surface, resulting in the loss of demineralized enamel structure [34, 35]. Fluoride toothpaste has a relatively low fluoride content and a small amount of usage per time. Lussi [36] found that when the concentration of fluoride ion is low (under 100 mg/L), fluorapatite was formed, which improves the acidity resistance of enamel. When the concentration of fluoride ion is higher, it combines with calcium ion to form calcium fluoride, promoting reminealization. The fluoride content in pea-sized fluoride toothpaste used for brushing teeth was about 2-3 mg, gargling after brushing further reduces the fluoride content that can bind to the enamel surface, so the therapeutic effect is weak. Uysal and other scholars found that brushing with fluoride toothpaste alone cannot prevent the progression of white spots lesions found during orthodontic treatment [37]. Hence, added remineralization treatments were needed when WSls were found.

The combined use of fluoride varnish plus F toothpaste and combined use of CPP-ACP plus F toothpaste showed the same remineralizing effect after 1 month. Fluoride varnish formed a thin layer of varnish firm which was stuck to the enamel surface after application, but because of the movement of buccal muscle, tongue, mastication, saliva wash, and oral hygiene practice, fluoride varnish is likely to be removed in a short time period due to the complicated oral environment and movement. It has been reported that fluoride varnish only remains in situ for up to 24 hours [38]. In this experiment, the mechanical friction caused by tooth brushing leads to the gradual stripping of Duraphat film. After 1 week, the varnish coat was unevenly exfoliated; after 1 month, it was completely removed mostly. According to the clinical research [12, 28, 29], the usage frequency of Duraphat treating WSLs is every 6 months, so fluoride ion could not be recharged on time, which led to the weakening of remineralizing effect. Hence, its efficacy fell dramatically compared with CPP-ACP group after 3 months.

CPP-ACP agent was used twice a day and held for half an hour after application; it was reported that the ion release profile of MI Paste plus was the most prominent among dental varnishes [39], Hence, the demineralized area could receive intermittent, high-frequency and high quantity of calcium, phosphate, and fluoride ions, so after 3 months, it had the best remineralizing effect.

Under SEM, a unit of structure which contains prism, interprismatic substance, and enamel sheath could be observed. Hydroxyapatite crystals are the main components of enamel, and these crystal fibers are arranged together, forming the enamel prism and the interprismatic substance [32]. Research [40] suggested that the composition of the prism and the interprismatic substance is the same, and the only difference was the orientation. Fibers which are close to the core are parallel to the long axis of prism; as they move away from the prism long axis, the orientation begins to deflect: the new orientation is perpendicular to the Retzius lines. This difference leads to discontinuity of the structure at their junctions, forming a gap, which is called the enamel sheath, and the main fillings of the enamel sheath are residual organic matrix degradation and saliva organic matter remained after enamel development and maturation [41].

After demineralization, dissolution of the enamel sheath caused shallow elliptical gap on the enamel surface. Pitted clefts due to the small amount of dissolution in the prism were invisible in the mild demineralized group; for the severe demineralized specimens, enamel structure dissolved notably, forming lager and deeper pores, as wells as clefts in the prism. These changes were in accordance with Diedirch's finding [42]: the initial stage of enamel demineralization took place around the prism head, causing micro clefts, which was due to the dissolution of the crystals in the enamel sheath and the sides. At the same time, the crystalline structure of prism became apparent with micropore. In the further stage, with the massive loss of enamel crystals, marginal clefts continued to widen, and pores in the prism became deep and wide. After remineralizing treatment by fluoride varnish and CPP-ACP agent, the deposition of newly formed minerals made the original rough and porous surface smooth and flattened, andpores and clefts became shallower because of the remineralization effect.

Treatment duration was set for 1 month and 3 months. 1 month is the common visit interval for orthodontic patients; it is the shortest time when orthodontists could reassess the lesions' condition as well as the treatment effect. Teenagers may have relatively poor compliance in hygiene and diet controls, but they are the major group of orthodontic patients. 3 months are a longer period, which might be a challenge for the implementation of added treatment procedure as some of the patients are reluctant to brush teeth standardly.

The enamel of the severe WSL group was damaged more seriously compared with the mild WSL group. Indicators of the severe group after 3 months of remineralization still could not compare with that of the mild demineralized specimens. These results suggested the importance of detecting white spot lesions in the early stage. Since visual examination is the most common detection method, orthodontists should examine the tooth surface after air-drying for 5 seconds carefully. Predilection site like the gingival side of the bracket needs to pay extra attention for both orthodontists during examination and patients during oral hygiene practice.

After 3 months of treatment, mild lesions were not fully recovered, and indicators and surface morphology were different from the sound enamel. For teenage patients with a high incidence of WSLs, it is often difficult for them to cooperate with additional measures. Hence, preventative method should be stressed in order to decrease the occurrence of WSLs, for example, providing targeted oral hygiene instruction through plaque staining and regular periodontal scaling.

Further study may be carried out on whether enamel could fully recover from the lesions and the treatment duration it would take. Widely used commercial products were selected in the experiment. With the development of novel materials, we look forward to safer and more effective agents to fight WSLs.

## 5. Conclusions

Based on the results, the following conclusions can be made:

(1) Mild and severe white spot lesions based on ICDAS were different in the degree of mineralization. Severe WSLs demineralized much more seriously than mild lesions even after 3 month's treatment.

(2) Despite the variation in severity, both lesions had the same variation trend after each treatment was applied: fluoride toothpaste had weak therapeutic effect and combined use of fluoride varnish plus F toothpaste and combined use of CPP-ACP plus F toothpaste were effective for remineralization. Their remineralizing efficacy was similar after 1 month, and combined use of CPP-ACP plus F toothpaste was more effective after 3 months.

## Data Availability

The data used to support the findings of this study are available from the corresponding author upon request.
